# Prospective blinded evaluation of smartphone-based ECG for differentiation of supraventricular tachycardia from inappropriate sinus tachycardia

**DOI:** 10.1007/s00392-021-01856-5

**Published:** 2021-05-07

**Authors:** Felix K. Wegner, Simon Kochhäuser, Gerrit Frommeyer, Philipp S. Lange, Christian Ellermann, Patrick Leitz, Patrick Müller, Julia Köbe, Lars Eckardt, Dirk G. Dechering

**Affiliations:** 1 Department of Cardiology II—Electrophysiology, University Hospital Muenster, Albert-Schweitzer-Campus 1, 48149 Muenster, Germany; 2 Present Address: Department of Cardiology and Vascular Medicine, West German Heart and Vascular Center Essen, University Duisburg-Essen, Essen, Germany; 3 Present Address: Department of Cardiology, Niels-Stensen-Kliniken Marienhospital Osnabrück, Osnabrück, Germany

**Keywords:** Supraventricular tachycardia, Inappropriate sinus tachycardia, Smartphone, AliveCor Kardia, ECG, Wearable, Digital medicine

## Abstract

**Introduction:**

Supraventricular tachycardias (SVT) are often difficult to document due to their intermittent, short-lasting nature. Smartphone-based one-lead ECG monitors (sECG) were initially developed for the diagnosis of atrial fibrillation. No data have been published regarding their potential role in differentiating inappropiate sinus tachycardia (IST) from regular SVT. If cardiologists could distinguish IST from SVT in sECG, economic health care burden might be significantly reduced.

**Methods:**

We prospectively recruited 75 consecutive patients with known SVT undergoing an EP study. In all patients, four ECG were recorded: a sECG during SVT and during sinus tachycardia and respective 12-lead ECG. Two experienced electrophysiologists were blinded to the diagnoses and separately evaluated all ECG.

**Results:**

Three hundred individual ECG were recorded in 75 patients (47 female, age 50 ± 18 years, BMI 26 ± 5 kg/m^2^, 60 AVNRT, 15 AVRT). The electrophysiologists’ blinded interpretation of sECG recordings showed a sensitivity of 89% and a specificity of 91% for the detection of SVT (interobserver agreement *κ* = 0.76). In high-quality sECG recordings (68%), sensitivity rose to 95% with a specificity of 92% (interobserver agreement of *κ* = 0.91). Specificity increased to 96% when both electrophysiologists agreed on the diagnosis. Respective 12-lead ECG had a sensitivity of 100% and specificity of 98% for the detection of SVT.

**Conclusion:**

A smartphone-based one-lead ECG monitor allows for differentiation of SVT from IST in about 90% of cases. These results should encourage cardiologists to integrate wearables into clinical practice, possibly reducing time to definitive diagnosis of an arrhythmia and unnecessary EP procedures.

**Graphical abstract:**

A smartphone-based one lead ECG device (panel **A**) can be used reliably to differentiate supraventricular tachycardia (panel **B**) from inappropriate sinus tachycardia when compared to a simultaneously conducted gold-standard electrophysiology study (panels **C**, **D**).
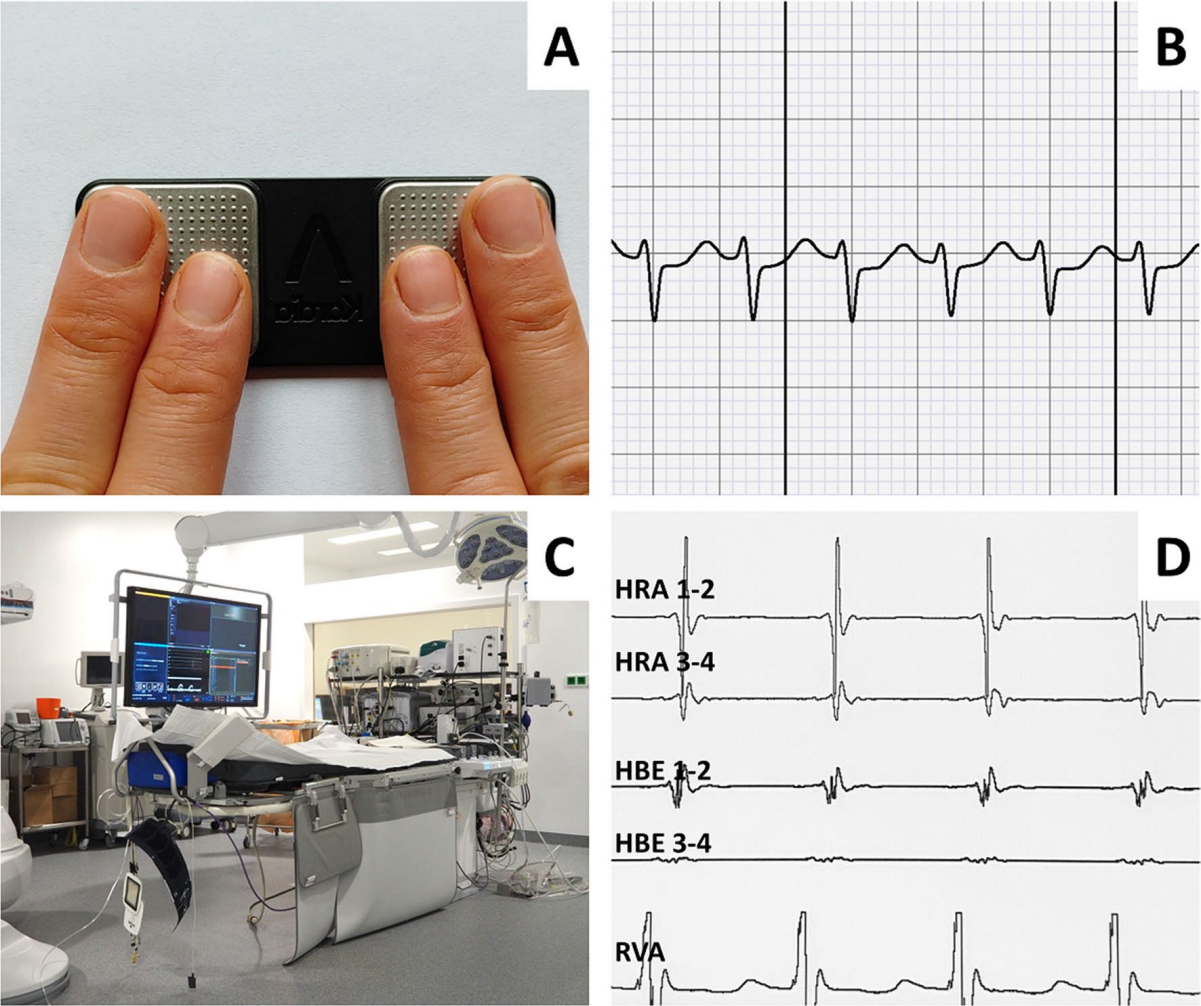

## Introduction

Supraventricular tachycardias (SVT) such as AV-nodal reentry tachycardia (AVNRT) and AV-reentry tachycardia (AVRT) are common etiologies of symptomatic regular tachycardia and can lead to significant subjective distress [1]. Ablation during an electrophysiology (EP) study is the curative first-line treatment [2, 3]. Inappropriate sinus tachycardia (IST) is an important differential diagnosis not amenable to invasive treatment in the majority of cases. The recording of an ECG during a symptomatic episode, therefore, remains the cornerstone of a correct diagnosis in suspected SVT [4]. However, due to often short-lasting symptoms, patients frequently struggle to reach a health-care setting in time for the recording of an ECG. This leaves the treating physician with the choice to either conduct an empirical EP study, even in patients with IST, or to have the patient continue trying to record the symptomatic tachycardia. Smartphone-based one-lead ECG devices (sECG) might be useful to quickly record the underlying rhythm, removing the need for an EP study when IST is documented. This would eliminate possible risks of invasive EP studies [5] and could have a beneficial impact on overall health care expenditure with optimized access to appropriate treatment. We, therefore, initiated this unsponsored, investigator-driven study with the FDA-cleared AliveCor Kardia sECG to test the hypothesis that the quality of an sECG recording would be sufficient to differentiate SVT from IST.

## Methods

### Study design

The present prospective single-center study was conducted in accordance with the Declaration of Helsinki. All patients gave informed consent to be included. We recruited 75 consecutive inpatients receiving an EP study for ECG-documented SVT at our center. In each patient, we recorded four intraprocedural ECG: sECG recordings of both the SVT and a sinus tachycardia during orciprenaline challenge (0.25 mg bolus, in case of only mild heart rate response repeated 0.25 mg bolus) and simultaneous 12-lead ECG counterparts. The AliveCor Kardia ECG device, originally designed and validated for the diagnosis of atrial fibrillation [6–8], was used for the recording of all sECG. All recordings were obtained from a consciously sedated patient during EP study. In case of deeper sedation, one of the authors helped to put both index fingers of the patient on the recording stick of the sECG. Recordings of atrial tachycardia were excluded from the study.

### Blinded interpretation

All ECG were anonymized, digitized and interpreted by two blinded experienced electrophysiologists. The electrophysiologists were asked to classify the recordings as either SVT or sinus tachycardia and adjudicate quality and interpretability on an ordinal scale (1 is not interpretable—5 is excellent quality) according to (a) signal to noise ratio, (b) discernibility of atrial activation, and (c) reproducibility of findings throughout the ECG. According to these metrics, recordings were defined as high, intermediate, or low quality. The respective interpretations of the sECG and the 12-lead ECG were compared to the definitive underlying rhythm as confirmed by intracardiac electrograms and diagnostic maneuvers described below. We calculated sensitivity and specificity for both the sECG and the 12-lead ECG.

### Electrophysiological study

In each patient, a routine EP study was conducted in a previously reported manner [9]. In short, we placed three diagnostic catheters via the femoral vein: in the apex of the right ventricle [right ventricular apex (RVA)], at the bundle of His (HIS) and in the high right atrium (HRA). Especially when an accessory pathway was suspected, a multipolar diagnostic catheter was placed in the coronary sinus (CS). Atrial and ventricular programmed stimulation was carried out down to a minimum cycle length of 330 ms with up to two additional beats (S3). In case of non-inducibility, we administered a bolus of orciprenaline (0.25 mg). When a sustained supraventricular tachycardia was induced, a 12-lead ECG was recorded simultaneously with a sECG. All patients were treated with radiofrequency ablation according to established practice and guideline recommendations [4]. During orciprenaline challenge after the ablation procedure, a further 12-lead ECG and an sECG of a sinus tachycardia were recorded.

### Statistical analysis

All digitized ECG were stored on a secure server. SPSS Version 25 was used for statistical analysis. Student’s *t* test was used for comparison of means. Mann–Whitney *U* test was utilized for comparison of numerical variables without a normal distribution. Fisher exact test or Chi-square test was used for comparison of categorical or binary variables. Interobserver agreement was measured using Cohen’s kappa. A two-sided *p* value of < 0.05 was considered statistically significant.

## Results

Forty seven of the 75 patients (63%) were female, the mean age of the patient population was 50 ± 18 years and the mean BMI was 26 ± 5 kg/m^2^. In 60 patients, a typical slow–fast AVNRT was the underlying SVT etiology. The remaining 15 patients exhibited an AVRT with only retrograde conduction properties of the accessory pathway. Accessory pathways were located posteriorly in ten patients, parahisian in two patients, midseptal in two patients and inferoseptal in one patient.

### Smartphone—ECG

One hundred and fifty sECG (75 SVT and 75 IST) were interpreted by the two electrophysiologists, resulting in 300 individual sECG interpretations. Table 1 depicts a cross tabulation of the blinded electrophysiologists’ diagnosis of all sECG compared to the gold-standard invasively measured rhythm during EP study. This resulted in a sensitivity of 89% and a specificity of 91% in the detection of SVT on sECG recordings. Interobserver agreement was substantial (*κ* = 0.76). Both blinded electrophysiologists reached agreement regarding the sECG diagnosis in 64 of 75 (85%) SVT recordings and 68 of 75 (91%) sinus tachycardia recordings (Table 1). Sensitivity in the detection of SVT was 95% and specificity increased to 96% when both electrophysiologists agreed on the diagnosis.

**Table 1:** Electrophysiologists’ blinded diagnosis of sECG and 12-lead ECG recordings compared to the underlying rhythm as established by EP study

		Underlying rhythm							
		All sECG		sECG with interobserver agreement		High-quality sECG		12-lead ECG	
		SVT (*n* = 150^a^)	Sinus tachycardia (*n* = 150^a^)	SVT (*n* = 128^a^)	Sinus tachycardia (*n* = 136^a^)	SVT (*n* = 105^a^)	Sinus tachycardia (*n* = 99^a^)	SVT (*n* = 150^a^)	Sinus tachycardia (*n* = 150^a^)
Electrophysiologists’ interpretation	SVT	133	13	122	6	100	8	150	2
	Sinus tachycardia	17	137	6	130	5	91	0	148
Sensitivity		89%		95%		95%		100%	
Specificity		91%		96%		92%		99%	

*sECG* smartphone-based one-lead ECG

^a^Numbers denote individual ECG interpretations by the two electrophysiologists, not the number of included patients (*n* = 75)

### High-quality smartphone—ECG

**Fig.1:**
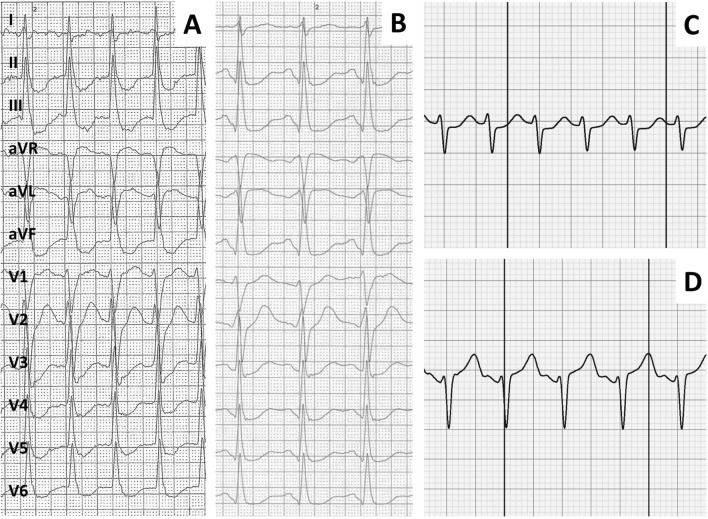
Depiction of SVT 12-lead ECG (**A**), sinus tachycardia 12-lead ECG (**B**), SVT sECG (**C**) and sinus tachycardia sECG (**D**) of the same patient in direct comparison. All ECG were correctly interpreted by both electrophysiologists and were described as high quality. Note that 12-lead ECG are written in 50 mm/s, while the sECG recordings are 25 mm/s

**Fig. 2:**
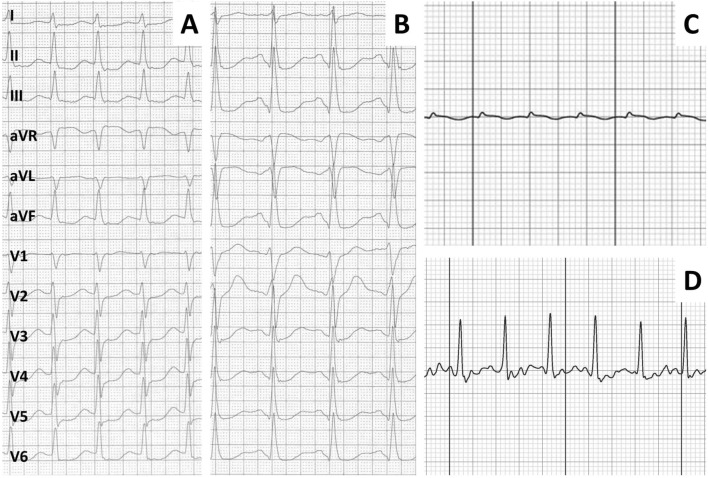
Comparison of correctly interpreted 12-lead ECG with incorrectly interpreted low-quality sECG. Panels **A** and **C** show an SVT of the same patient on 12-lead ECG and sECG, respectively. Panels **B** and **D** show a sinus tachycardia in a different patient. While the electrophysiologists agreed on the diagnosis in panels **A** and **B**, there was disagreement as to the diagnosis in panels **C** and **D**. Writing speed for the 12-lead ECG is 50 mm/s and for the sECG 25 mm/s

Two hundred and four (68%) sECG interpretations described the underlying recording as high quality. Table 1 shows a cross tabulation of the blinded electrophysiologists’ diagnosis compared to the definitive diagnosis. In these high-quality recordings, the sensitivity and specificity of the sECG increased to 95% and 92%, respectively, with an almost perfect interobserver agreement (*κ* = 0.91). Representative high-quality sECG recordings which were correctly interpreted are exhibited in Fig. 1, while Fig. 2 shows medium and low quality sECG recordings which were in these cases not consistently interpreted by the electrophysiologists.

### Twelve-lead ECG

**Fig. 3:**
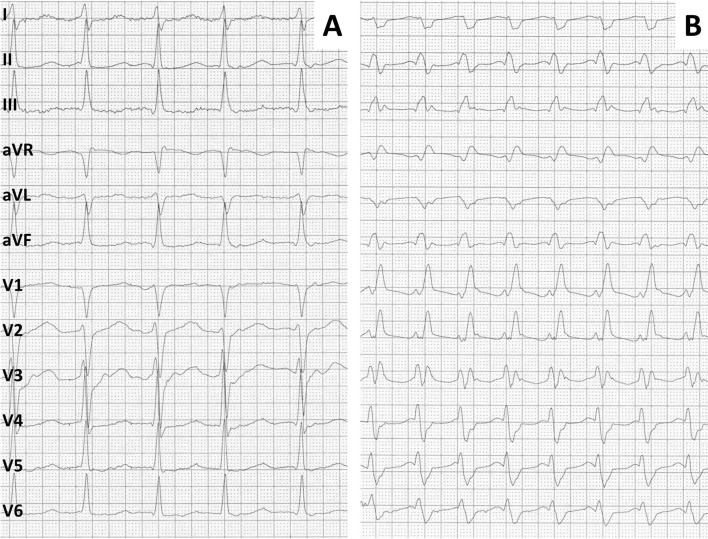
Depiction of the two 12-lead SVT ECG being misinterpreted as sinus tachycardia by one of the two analyzing electrophysiologists. Writing speed is 50 mm/s

In all patients, 12-lead ECG of the SVT and sinus tachycardia were obtained simultaneously to the respective sECG recordings. A cross tabulation of the diagnosis of the 12-lead ECG recordings and the corresponding EP study-based diagnosis is shown in Table 1. The 12-lead ECG had a sensitivity of 100% in the detection of an SVT with a specificity of 99%. Interobserver agreement was *κ* = 0.97. The two 12-lead ECGs that were misinterpreted by one of the electrophysiologists are shown in Fig. 3.

### Sensitivity analysis

The median SVT cycle length was 350 ms (interquartile range 300–400 ms) and the median sinus tachycardia cycle length was 400 ms (interquartile range 360–480 ms). The difference between SVT and sinus tachycardia cycle length was not associated with an increased likelihood of a correct sECG diagnosis (*p* = 0.52). To expand on this, we conducted a sensitivity analysis in a subgroup of 25 patients whose SVT and IST cycle lengths were between 300 and 500 ms and within 50 ms of each other in each individual patient. In this subgroup, diagnostic accuracy of the sECG was not altered with a sensitivity of 88% and a specificity of 92%. Furthermore, the underlying SVT type (AVNRT vs AVRT) was not associated with a higher likelihood of a correct diagnosis (*p* = 0.68). Additionally, neither gender (*p* = 0.10), age (*p* = 0.46), nor BMI (*p* = 0.87) were predictive of a correct sECG diagnosis.

## Discussion

The present unsponsored, investigator-initiated study is the first to report on the diagnostic utility of a smartphone-based one-lead ECG recorder for the diagnosis of regular supraventricular tachycardia. We were able to demonstrate high accuracy of the sECG in the detection of SVT when compared to the gold-standard invasive EP procedure.

Our studied population of consecutive patients closely resembles a standard SVT population in gender and age distribution. While many patients present at a relatively young age, SVT can develop at any stage of life. We were able to show that sECG reliably record the underlying rhythm in paroxysmal tachycardia regardless of the age of the affected patient or other possible signal influencing factors such as gender or BMI.

Smartphone-based ECG recording devices have previously been evaluated for the diagnosis of atrial fibrillation and were shown to reliably document the underlying rhythm [6–8]. Subsequently, one-lead ECG devices such as the AliveCor Kardia and the Apple Watch have been increasingly used by patients to document symptomatic tachycardias [10]. However, concerns seem to persist among cardiologists: in a recent European survey, only half of respondents would have performed an EP study when sECG showed (a) a narrow complex tachycardia in a patient with (b) typical on/off palpitations [11]. In this regard, the present study might close an important gap by establishing that regular SVTs can be differentiated from inappropriate sinus tachycardia in sECG with a high accuracy, which holds especially true in high-quality sECG.

With exponentially growing medical expenditures, there is urgent need to reduce global economic burden on our health care systems. One of the promising strategies is digital medicine to make processes more efficient. Out of necessity, during the current COVID-19 pandemic, digitalization of medical processes has been hugely intensified. While the 12-lead ECG remains the most accurate noninvasive diagnostic test to distinguish SVT from sinus tachycardia, our data may help clinicians integrate sECG into clinical practice in suspected SVT, thereby promoting the transfer to digital medicine.

Sensitivity (89%) and specificity (91%) for the detection of SVT were comparable with previously reported sensitivity (90–100%) and specificity (89–97%) for the detection of atrial fibrillation [6–8]. The diagnostic utility and interobserver agreement considerably increased when sECG were high quality, highlighting the need for patient instruction on how to record episodes. Strategies to reduce artifact in recorded ECG may include constant pressure of the fingers on the sECG recorders, no movement of fingers while recording, the use of different fingers, small amounts of water to reduce electrical impedance or the use of alternative recording vectors [7, 12]. Unsurprisingly, specificity for SVT detection increased when two electrophysiologists agreed on the underlying rhythm.

This is the first study to compare sECG recordings to the gold-standard EP study. We were thus able to directly compare the diagnostic yield of the sECG to a 12-lead ECG, which showed an equivocal recording in rare cases (see Fig. 3). While the differentiation of atrial fibrillation from sinus rhythm can be achieved even when there is a considerable amount of artifact in the recording by analyzing the regularity of R–R intervals, distinguishing SVT from IST, at least for now, requires interpretation of the whole ECG by a human specialist (see Figs. 1, 2), as both SVT and IST typically present with a tachycardia with regular R–R intervals. We were able to demonstrate that sECG recordings can—with human interpretation—reliably be employed even in this higher demand setting and diagnostic accuracy does not relevantly diminish in comparison with the diagnosis of atrial fibrillation. Whether machine learning/artificial intelligence can reliably interpret narrow complex sECG in the near future remains to be seen.

In everyday electrophysiology practice, patients regularly present with a history of highly symptomatic paroxysmal tachycardias suggestive of SVT without ECG documentation. Our data show that smartphone-based one-lead ECG recordings can be utilized in suspected SVT and may, therefore, reduce the time to definitive diagnosis and a potential cure in the form of EP-guided ablation. Furthermore, should the underlying rhythm be inappropriate sinus tachycardia, a potentially unnecessary invasive EP study can be avoided, reducing possible distress and complications for the individual and decreasing overall economic healthcare burden.

## Limitations

The present results are not applicable to all SVT as it would be very difficult to distinguish atrial tachycardia from sinus tachycardia in a single-lead ECG. However, as atrial tachycardias comprise only about 5–10% of patients [13, 14], there would only be a small fraction of SVT patients, where a differentiation would not be possible. Recorded SVT had a shorter median cycle length than recorded sinus tachycardias. While this may have allowed the electrophysiologists to distinguish between the recordings more easily (the faster the narrow complex tachycardia, the more likely the diagnosis is SVT) the difference in cycle length did not affect the likelihood of a correct diagnosis, making confounding by cycle length unlikely. Additionally, by recording sinus tachycardia and SVT in the same patients we were able to considerably reduce the possible confounder of inter-individual sECG recording quality. Furthermore, we would like to stress the importance of patient instruction in how to record a high-quality sECG, since insufficient recording quality may remain as the predominant factor in preventing the widespread use of sECG.

## Conclusion

A smartphone-based one-lead ECG can reliably differentiate SVT from IST, especially in high-quality recordings. This highlights the need to instruct patients how to optimally use wearables to increase the diagnostic yield. Our analyses also indicate that in difficult cases, consulting a colleague increases the likelihood of a correct ECG diagnosis. The results of this study hopefully encourage cardiologists to implement digital medicine in their workflow of diagnosing several cardiac arrhythmias, not only atrial fibrillation.
